# Clinical implementation of pre-treatment *DPYD* genotyping in capecitabine-treated metastatic breast cancer patients

**DOI:** 10.1007/s10549-019-05144-9

**Published:** 2019-02-12

**Authors:** Chara Stavraka, Athanasios Pouptsis, Leroy Okonta, Karen DeSouza, Philip Charlton, Matthaios Kapiris, Anthony Marinaki, Eleni Karapanagiotou, Dionysis Papadatos-Pastos, Janine Mansi

**Affiliations:** 1 Breast Unit, Guy’s and St Thomas’ NHS Foundation Trust and King’s Biomedical Centre, 4th Floor, Bermondsey Wing, Great Maze Pond, London, SE1 9RT UK; 2 Purine Research Laboratory, Viapath, Guy’s and St Thomas’ NHS Foundation Trust, Westminster Bridge Road, London, SE1 7EH UK

**Keywords:** DPYD genotyping, DPYD screening, Fluoropyrimidines, Toxicities, Metastatic breast cancer, Capecitabine

## Abstract

**Purpose:**

Metastatic breast cancer (mBC) patients with *DPYD* genetic variants linked to loss of dihydropyrimidine dehydrogenase (DPD) activity are at risk of severe capecitabine-associated toxicities. However, prospective *DPYD* genotyping has not yet been implemented in routine clinical practice. Following a previous internal review in which two patients underwent lengthy hospitalisations whilst receiving capecitabine, and were subsequently found to be DPD deficient, we initiated routine *DPYD* genotyping prior to starting capecitabine. This study evaluates the clinical application of routine *DPYD* screening at a large cancer centre in London.

**Methods:**

We reviewed medical records for consecutive patients with mBC who underwent DPYD genotyping before commencing capecitabine between December 2014 and December 2017. Patients were tested for four *DPYD* variants associated with reduced DPD activity.

**Results:**

Sixty-six patients underwent *DPYD* testing. Five (8.4%) patients were found to carry *DPYD* genetic polymorphisms associated with reduced DPD activity; of these, two received dose-reduced capecitabine. Of the 61 patients with *DPYD* wild-type, 14 (23%) experienced grade 3 toxicities which involved palmar–plantar erythrodysesthesia (65%), and gastrointestinal toxicities (35%); no patient was hospitalised due to toxicity.

**Conclusions:**

Prospective *DPYD* genotyping can be successfully implemented in routine clinical practice and can reduce the risk of severe fluoropyrimidine toxicities.

## Introduction

Breast cancer is the most common malignancy among women accounting for 23% of new cancer cases and 15% of cancer-related deaths annually in the world [1]. Capecitabine, an orally administered pro-drug of 5-fluoruracil has a pivotal role in the therapeutic armamentarium for metastatic breast cancer (mBC) usually as monotherapy but also in combination with lapatinib [2]. Fluoropyrimidines are considered to be safe and well tolerated with a side-effect profile that includes diarrhoea, mucositis, palmar–plantar erythrodysesthesia (PPE), bone marrow suppression and nausea. Although the majority of patients receiving fluoropyrimidines experience mild toxicities, usually managed with supportive measures, approximately 10–30% of patients will develop severe (grade ≥ 3) toxicities which can result in lengthy hospitalisations or death in 0.5–1% of cases [3–5].

Fluoropyrimidine toxicities have been strongly associated with reduced activity of the enzyme dihydropyrimidine dehydrogenase (DPD), which is the rate-limiting enzyme in the catabolism of fluoropyrimidines [6–9]. DPD catabolises 80% of 5-fluoruracil (5-FU), into the non-cytotoxic metabolite 5-fluoro-5,6-dihydrouracil (dHFU) and patients with partial deficiency have 1.5-times increased 5-FU exposure when treated with standard doses [6, 10]. Approximately, 3–5% of the North American and European population have partial DPD deficiency, whereas complete deficiency is much rarer with a prevalence of 0.01–0.1% [11–13].

The activity of DPD has been found to be regulated at the genetic, transcriptional (by transcription factors SP1 and AP3) and post-transcriptional level (microRNA 27-a and microRNA 27-b) [14–16]. The most prevalent cause of DPD deficiency though, is the presence of deleterious polymorphisms in its encoding *DPYD* gene, which have received much interest as predictive biomarkers for fluoropyrimidine-induced toxicities [13, 17–21]. Four *DPYD* genetic variants have been established as clinically relevant and associated with severe toxicities: DPYD*2A (IVS14 + 1G > A, c.1905 + 1G > A, or rs3918290), c.2846A > T (p.D949V or rs67376798), c.1679T > G (rs55886062, DPYD*13, p.I560S), and c.1236G > A (rs56038477, p.E412E, Hap B3) [13, 22–25]. A fifth variant c.1601G > A (p.S534N, DPYD*4, or rs1801158), has been associated with impaired DPD activity [26] but its clinical relevance remains inconclusive [13]. Current evidence suggests that heterozygous carriers of these variants have an average decrease in the DPD activity of approximately 25% (c.2846A > T, c.1236G > A) and 50% (DPYD*2A, c.1679T > G) [24]. The Clinical Pharmacogenetics Implementation Consortium (CPIC) has issued guidance on appropriate dose reductions in patients harbouring these polymorphisms to prevent severe toxicities [24] Despite growing evidence supporting the significant clinical and financial benefits of routine *DPYD* genotype screening prior to fluoropyrimidine administration, this is not yet the standard of care [27–32]. The ability to predict and prevent severe capecitabine-associated toxicities is of paramount importance particularly in metastatic breast cancer patients, as a number of alternative treatment options are available. Such toxicities can result in death or pose significant delays to consequent treatment and may even compromise patients’ fitness to receive further treatment.

Based on our initial experience with two mBC patients who were hospitalised and retrospectively found to be DPD deficient, we previously conducted an internal review including 48 patients and found that: (a) none of the other patients who were receiving capecitabine during that time (December 2012 to December 2013) underwent lengthy hospital admission for capecitabine toxicities and (b) the cost of the inpatient stay far outweighed the total cost of testing all those patients for DPD (£15 525 versus £1575 based on the cost of the test at that time) [33].

Following this, routine *DPYD* screening prior to prescribing capecitabine was initiated at Guy’s and St Thomas’ NHS Trust (GSTT) in December 2014 using an in-house developed genotyping assay [34]. In this retrospective observational study, we describe our centre’s experience and clinically evaluate prospective DPYD genotyping for metastatic breast cancer patients treated with capecitabine. In particular, we evaluated the feasibility of implementing DPYD tests in routine clinical practice, treatment decisions and outcomes based on the pharmacogenetic test results, and its utility in preventing severe toxicities.

## Methods

### Prospective DPYD genotyping

At GSTT routine, *DPYD* genotype screening was implemented in December 2014 for all mBC patients who were thought suitable to receive capecitabine. Patients were tested for the following four *DPYD* genetic variants which are prevalent in the British population and associated with severe capecitabine-associated toxicities: DPYD*2A (IVS14 + 1G > A, c.1905 + 1G > A, or rs3918290), c.2846A > T (p.D949V or rs67376798), c.1679T > G (rs55886062, DPYD*13, p.I560S) and c.1601G > A (S534N, DPYD*4, or rs1801158) [34, 35]. The pharmacogenetic tests were performed in our institutional laboratory and their prospective nature was determined by comparing the genotyping date with the date of treatment initiation.

### Patient population

We retrospectively identified metastatic breast cancer patients who were prescribed capecitabine (monotherapy or in combination with lapatinib) between December 2014 and December 2017 at GSTT.

Electronic patient records were reviewed, and information collected on demographic and clinical characteristics including age, gender, ECOG performance status, prescribed treatment regimen, performance of pre-treatment *DPYD* genotyping test, *DPYD* genotype outcome, time of treatment initiation, nature and grade of capecitabine-associated toxicities, dose modifications, reasons for treatment discontinuation and hospitalisation. Toxicities were graded according to the NCI—CTCAE v4.0. The feasibility of the routine application of *DPYD* pre-treatment testing in clinical practice was evaluated by assessing the percentage of patients screened for *DYPD* when a prescription of capecitabine was given. The turnaround time of the test was also evaluated as well as the presence of associated treatment delays. Patients who did not receive capecitabine due to non *DPYD*-related reasons were not included in the analysis.

### Statistical analysis

Descriptive statistics were generated to characterise the study cohort in terms of clinicopathological parameters. Categorical outcomes were presented as a frequency and proportion. The SPSS statistical package version 25 (IBM SPSS Inc., USA).

Official approval for the use of retrospective data was granted by Guy’s and St Thomas’ Clinical Audit Office. Data were handled in accordance with the Declaration of Helsinki and all patients had provided informed consent prior to *DPYD* testing.

## Results

Between December 2014 and December 2017, a total of 72 consecutive mBC patients were considered for capecitabine as a monotherapy or in combination with lapatinib and tested for DPD deficiency. All patients had a pre-treatment *DPYD* test (100%). Of these 72 patients, 5 did not receive capecitabine due to poor performance status or disease complications, and 1 patient died 6 days after starting cycle 1 due to variceal bleeding. None of these six patients had a *DPYD* variant and they were excluded from the analysis. The final analysis was on the remaining 66 patients. Patient characteristics are summarised in Table 1. Five of 66 (8%) were found to be heterozygous variant allele carriers (Table 1) with their pharmacogenomic test results being accompanied by recommendations for dose modifications as per our institutional guidelines as follows: heterozygous c.1905 + 1G > A for treatment with 50% dose reduction, heterozygous c.2846A > T and heterozygous c.1601G > A for treatment with 25% dose reduction [34]. Three of these patients did not receive capecitabine, and an alternative regimen was prescribed by their treating physician. Treatment outcomes for the *DPYD* variant carriers are summarised in Table 2. The other two patients received a 50% dose reduction of capecitabine during their first cycle of treatment with no complications. The c.2846A < T variant carrier had a subsequent dose increase to 75% on cycle 2 which was tolerated very well. Conversely, on increasing the dose for the c.1905 + 1G > A carrier she developed grade 3 toxicities (PPE, diarrhoea, nausea, neutropaenia) requiring hospitalisation for 10 days and treatment cessation.

**Table 1:** Patient demographic and clinical characteristics

Characteristic	Number (%), *N* = 66
Sex	
Male	2 (3%)
Female	64 (97%)
Mean age, years	58 (28–85)
ECOG performance status	
0	22 (33%)
1	24 (36%)
2	9 (14%)
3	2 (3%)
Not specified	9 (14%)
DPYD status	
Wild type	61 (92%)
Heterozygous c.1601G > A	2
Heterozygous c.2846A < T	2
Heterozygous c.1905 + 1G > A	1
Capecitabine received	63 (95%)
Single agent	49 (22%)
Combination with lapatinib	14 (78%)

*ECOG* Eastern Cooperative Oncology Group Performance Status

**Table 2:** Treatment outcomes based on pharmacogenomic results for patients carrying DPYD polymorphisms

No.	Patient summary	DPYD variant	Treatment outcome
1	56F, ECOG PS1	Heterozygous c.2846A < T	Capecitabine not given Alternative treatment
2	42F, ECOG PS 2	Heterozygous c.1601G > A	Capecitabine not given Alternative treatment
3	62F, ECOG PS 1	Heterozygous c.1601G > A	Capecitabine not given Alternative treatment
4	45F, ECOG PS 0	Heterozygous c.1905 + 1G > A	C1 DR 50% with no complications, C2 dose increased to 75%: admitted with G3 toxicities (PPE, diarrhoea, nausea, neutropaenia), treatment stopped, and patient recovered
5	59F, ECOG PS 0	Heterozygous c.2846A < T	C1 DR 50%: no complications. C2 dose increased to 75%: no toxicities

*F* female, *ECOG PS* Eastern Cooperative Oncology Group Performance Status, *C* cycle, *DR* dose reduction

Of the 61 patients with a wild-type *DPYD* genotype, 14 (23%) experienced capecitabine-related adverse events (G > 3) such as PPE and gastrointestinal symptoms including diarrhoea and mucositis (Table 3). In two patients, treatment was stopped, however, none of these patients required hospitalisation. A total of 9 patients (15%) required a dose reduction at the outset of treatment due to comorbidities or poor performance status. There was no death associated with capecitabine treatment in our patient cohort.

**Table 3:** Capecitabine-related toxicities and dose modifications in DPYD wild-type patients

Toxicity ≥ grade 3	Number (%) (*N* = 61)
Overall toxicity	14 (23%)
Gastrointestinal toxicities	5 (8%)
Palmar-plantar erythrodysesthesia	9 (15%)
Haematological	0 (0%)
Capecitabine-related hospital admissions	0 (0%)
Treatment discontinuation because of capecitabine induced adverse events	2 (3%)
Capecitabine related deaths	0 (0%)
Dose reduction on treatment outset	
25%	6 (10%)
50%	3 (5%)

The test turnaround time for the *DPYD* genotyping results was 2–3 working days and did not cause delays in treatment initiation. The cost of the test was £57.56 per patient.

## Discussion

In this observational study, we evaluated the feasibility and usefulness of routine prospective *DPYD* genotyping for the prevention of severe toxicities in mBC patients treated with capecitabine. Our results show that the implementation of *DPYD* screening in clinical practice was feasible and well accepted by clinicians, as every patient who was considered for capecitabine in our institution was successfully screened. The rapid turnaround time and relatively low cost of the test contributed to this, although these factors may vary across different treatment centres globally. This is in keeping with outcomes from large prospective multicentre studies supporting the feasibility and cost-effectiveness of prospective *DPYD* genotyping for patients receiving fluoropyrimidine-based treatment across all tumour types [27, 28].

This evaluation is limited by its relatively small sample size. The low number of *DPYD* variant carriers precludes a formal evaluation of the effect of *DPYD* screening on capecitabine-induced toxicities.

Our patients were screened at our in-house facility for four *DPYD* variants with an assay that had a combined predictive value of > 99% and negative predictive value of 80% [34]. Only two of the five patients identified as carriers of *DPYD* polymorphisms received a reduced dose of capecitabine; this was 50% in both patients, but of note was the severe toxicity when the dose was increased by 25% for one of these patients. The consequence of giving full dose capecitabine, without the knowledge of the DPD deficiency, could have resulted in very severe morbidity or even death.

The patients who did not receive capecitabine carried c.2846A > T and c.1601G > A polymorphisms, for which a recommendation of 25% dose reduction is advised. The variability seen in the treatment decisions made by the physicians could reflect uncertainty due to the lack of adequate safety data in the literature at that time. Only recently have large studies emerged evaluating the clinical relevance and providing safety outcomes for commonly screened variants [13, 27, 28, 32]. Although the Clinical Pharmacogenetics Implementation Consortium (CPIC) has issued comprehensive guidance on dose adjustments, there is a need for more real-world safety data to identify the optimal dosing for each genotype [24, 28].

From the patients found to be wild-type *DPYD*, only 23% developed grade 3 toxicities which were managed with supportive measures or dose reductions but did not result in hospitalisation. It has been previously reported that patients who do not carry *DPYD* variants can still experience severe side effects. This can be due to the sensitivity of the assay, the presence of unrecognised and hence unscreened polymorphisms, or post-transcriptional modifications and variation in other genes influencing fluoropyrimidine drug metabolism [14, 15, 24, 28, 30]. DPD phenotyping testing can predict DPD activity more accurately than *DPYD* genotyping, however, its cost and lengthy turnaround time make it difficult to implement in clinical practice [36]. Similarly, not all patients carrying deleterious *DPYD* variants will experience severe toxicity at standard doses, which may result in their undertreatment. Careful dose titration upon an initial dose reduction has been suggested by CPIC to address this issue [24].

Despite the growing amount of evidence favouring the routine implementation of *DPYD* genotype screening in clinical practice [27, 28, 31], this remains a subject of debate and controversy. Utilising pharmacogenomics to prevent avoidable toxicities, and death, is of paramount importance for patient care. This has even greater implications for patients with mBC for whom a plethora of therapeutic options are available. Our study demonstrates that routine implementation of *DPYD* genotyping in this patient population is feasible and can guide treatment decisions in a personalised manner.

## Data Availability

The datasets during and/or analysed during the current study are available from the corresponding author on reasonable request.
